# Stop! Stop!

**Published:** 1913-04

**Authors:** 


### Stop! Stop!

It is too often a regrettable fact that in the advocacy of a cause there are some adherents who, from overzeal, miss the mark and seriously affect the main issue. The all-important and absorbing question of sex instruction has not proven an exception to this rule. It seems but yesterday that reticence was the unwritten law. Now behold the avalanche of promiscuous literature seeking to enlighten the world, seemingly awakening from the misty dream of misinformation. With the barrier of reticence broken, periodicals and publishing houses seem to vie one with the other in making these

"save the race" articles, shorn of the conventionalities usually observed in the presentation of such questions. It is needless to say that the mass of them are inaccurate, puerile and misdirected, not to say demoralizing.

Not one, however, will gainsay that the silence of our fathers was destructive to the welfare of the child. It is the daily lament of thousands: "If I had only known. My parents never spoke to me on such matters." The result is seen in the wrecked lives and homes of thousands and thousands of the flower of our civilization.

The question is not whether it is right or wrong to impart sex instruction, but when and how it should be done.

To do this properly requires a greater knowledge of child psychology, than the average parent or teacher possesses. The crucial purpose is to acquaint the child with such knowledge of the physiology of sex that will afford him protection from vice during the development of his mind and body until it has reached the period of full fruition. The stumbling block has been the danger of awakening in the child morbid curiosity about sex that would undermine all other teaching. To obviate this the moral nature must be developed first, and made the dominating element in the child's life, until he has arrived at his sex maturity. He must be led cautiously to see the naturalness and sacredness of the mystery of birth and to sanctify motherhood. He must be taught strength of character by learning to overcome obstacles, and resist temptations, as only by strength gained in this way can he hope to fortify himself for the pitfalls that will beset him on every hand. Above all, avoid pedagogic information, such as is illustrated in the instance of the seven-year-old boy of a professor in one of our leading universities. He entertained the visitors at his home by relating the circumstances in connection with the misfortune of his mother in receiving lacerations during childbirth. Such prodigiousness is shocking and misses the mark of the higher purpose of his sex instruction.

Nobler moral qualities are inherited as well as vicious ones, and if morality is to endure in the end there will have to be a more intentional application of the laws of eugenics.

